# Diffusion analysis of fluid dynamics with incremental strength of motion proving gradient (DANDYISM) to evaluate cerebrospinal fluid dynamics

**DOI:** 10.1007/s11604-020-01075-4

**Published:** 2021-01-02

**Authors:** Toshiaki Taoka, Hisashi Kawai, Toshiki Nakane, Takashi Abe, Rei Nakamichi, Rintaro Ito, Yuki Sato, Mayuko Sakai, Shinji Naganawa

**Affiliations:** 1 Department of Innovative Biomedical Visualization (iBMV), Nagoya University Graduate School of Medicine, 65 Tsurumai-cho, Showa-ku, Nagoya, Aichi 466-8550 Japan; 2 Department of Radiology, Nagoya University Graduate School of Medicine, 65 Tsurumai-cho, Showa-ku, Nagoya, Aichi 466-8550 Japan; 3 Canon Medical Systems Corporation, 1385 Shimoishigami, Otawara-shi, Tochigi 324-8550 Japan

**Keywords:** Diffusion-weighted image, Low *b* value, Diffusion analysis of fluid dynamics with incremental strength of motion proving gradient (DANDYISM), Cerebrospinal fluid dynamics

## Abstract

**Purpose:**

To visualize and analyze the dynamics of cerebrospinal fluid (CSF) motion in the cranium, we evaluated the distribution of motion-related signal dephasing by CSF on Diffusion ANalysis of fluid DYnamics with Incremental Strength of Motion proving gradient (DANDYISM) method, a composite imaging method using various low *b* values.

**Materials and methods:**

This study examined ten subjects aged 25–58. We acquired DWIs on a 3T clinical scanner with *b* values 0, 50, 100, 200, 300, 500, 700, and 1000 s/mm^2^ in total imaging time of 4 min. We constructed DANDYISM images and evaluated the CSF area distribution with decreased motion-dephasing signal using a scoring method.

**Results:**

The DANDYISM images showed statistically significant higher CSF scores in the ventral posterior fossa, suprasellar cistern, and Sylvian vallecula compared to the lateral ventricle and frontal and parietal CSF spaces, indicating greater CSF movement in the former areas.

**Conclusion:**

The results indicated prominent CSF motions in the ventral portion of the posterior fossa, suprasellar cistern, and Sylvian fissure but smaller motions in the lateral ventricles and parietal subarachnoid space. This method may provide information of CSF dynamics in the clinical settings within short imaging time.

## Introduction

The classical theory of cerebrospinal fluid (CSF) dynamics, which was established in the early twentieth century [1, 2], suggests that CSF is produced by the choroid plexus in the ventricle and flows out of the ventricular system from the foramens of Luschka/Magendie into the subarachnoid space in the surface of the brain, before being absorbed by the arachnoid granules distributed in the parasagittal area. The flow is considered to be river-like. However, many evidences have cast a doubt on this classical model of river-like CSF recently.

Diffusion-weighted images (DWIs), which visualize the motion of water molecules as a signal decreases due to a phase shift caused by the motion, can be used to study CSF dynamics. When the phase shift becomes larger than ± π or multiple velocities exist in a single voxel, the signal decrease in the DWI becomes prominent according to the b values (motion-related signal dephasing). Thus, CSF motion causes notable signal decrease due to its large or non-uniform motion [3, 4]. It has been reported that a DWI of *b* = 500 s/mm^2^ reflects changes in the CSF dynamics [5]. In the study, a DWI of *b* = 500 s/mm^2^ for CSF within the lateral ventricle showed a higher signal in the ventricle dilatation group than in the control group. However, a single b value such as *b* = 500 s/mm^2^ cannot provide the detailed distribution of the CSF dynamics. DWIs of various b values can visualize the degree of CSF motion. As shown above, although not quantitative, a DWI with a lower b value will show motion-related signal dephasing only in areas with larger CSF motion, whereas DWI with higher b value will show motion-related signal dephasing of CSF in a wider area with small to large CSF motion. We developed a composite imaging method using various low b values called “Diffusion ANalysis of fluid DYnamics with Incremental Strength of Motion proving gradient (DANDYISM)” for evaluating the distribution of areas with signal dephasing on DWIs of various b values.

The purpose of the current study is to evaluate CSF dynamics using DANDYISM, which is a composite imaging method using various low b value DWIs, and to evaluate the feasibility of this method to provide information of whole cranial CSF dynamics.

## Subjects and methods

**Fig. 1:**
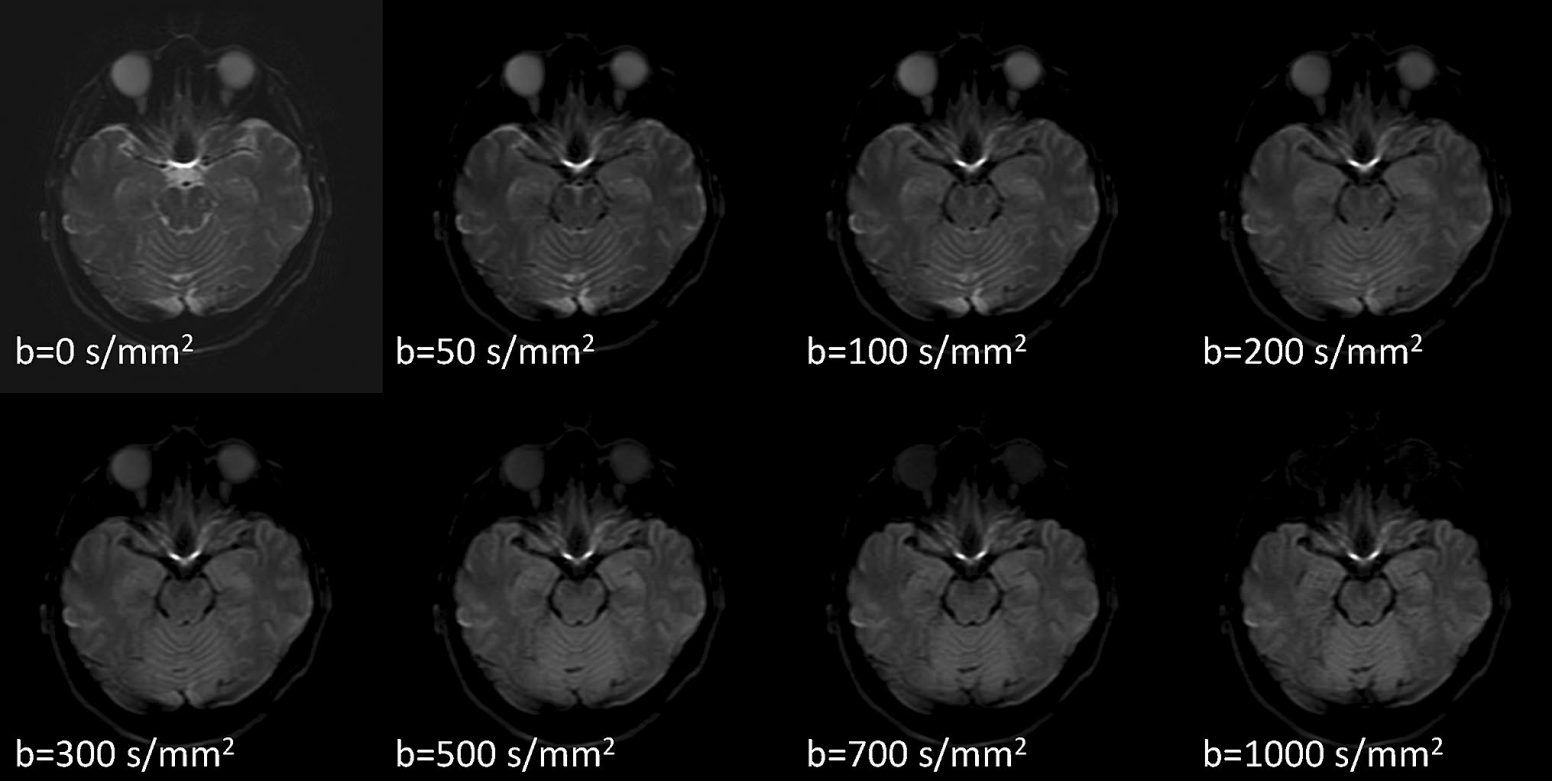
Diffusion-weighted images (DWIs) with various b values. As a motion-proving gradient with higher b value is applied, signal drop by motion-related signal dephasing occurs in the CSF, especially in the area with large CSF motions. Note that the area with signal drop in the CSF space increases in DWIs with higher b values

This study on DWIs for CSF dynamics has been approved by the institutional review board of our institution. The subjects of this study were ten volunteers, aged 25–58 years, without any abnormal findings in a conventional MRI brain scan; the subjects provided written informed consent. DWIs (Fig. 1) were acquired using a 3T clinical scanner (Vantage Centurian, Canon Medical Systems, Tochigi, Japan) with the following parameters: repetition time = 5000 ms, echo time = 85 ms, echoplanar imaging factor = 60, echo spacing = 0.7 ms, band width = 1953 Hz/Px, δ = 18.3 ms, Δ = 43.6 ms, field of view = 220 mm, 192 × 128, 25 slices with distance factor = 10%, slice thickness = 5 mm, averages = 2, acceleration factor = 3, acquisition time = 4 min for total acquisition, motion-probing gradient: monopolar type, *b* value = 0, 50, 100, 200, 300, 500, 700, 1000 s/mm^2^, 3-axis mixed, with fat suppression, no flow compensation, no motion correction, no distortion correction.

**Fig. 2:**
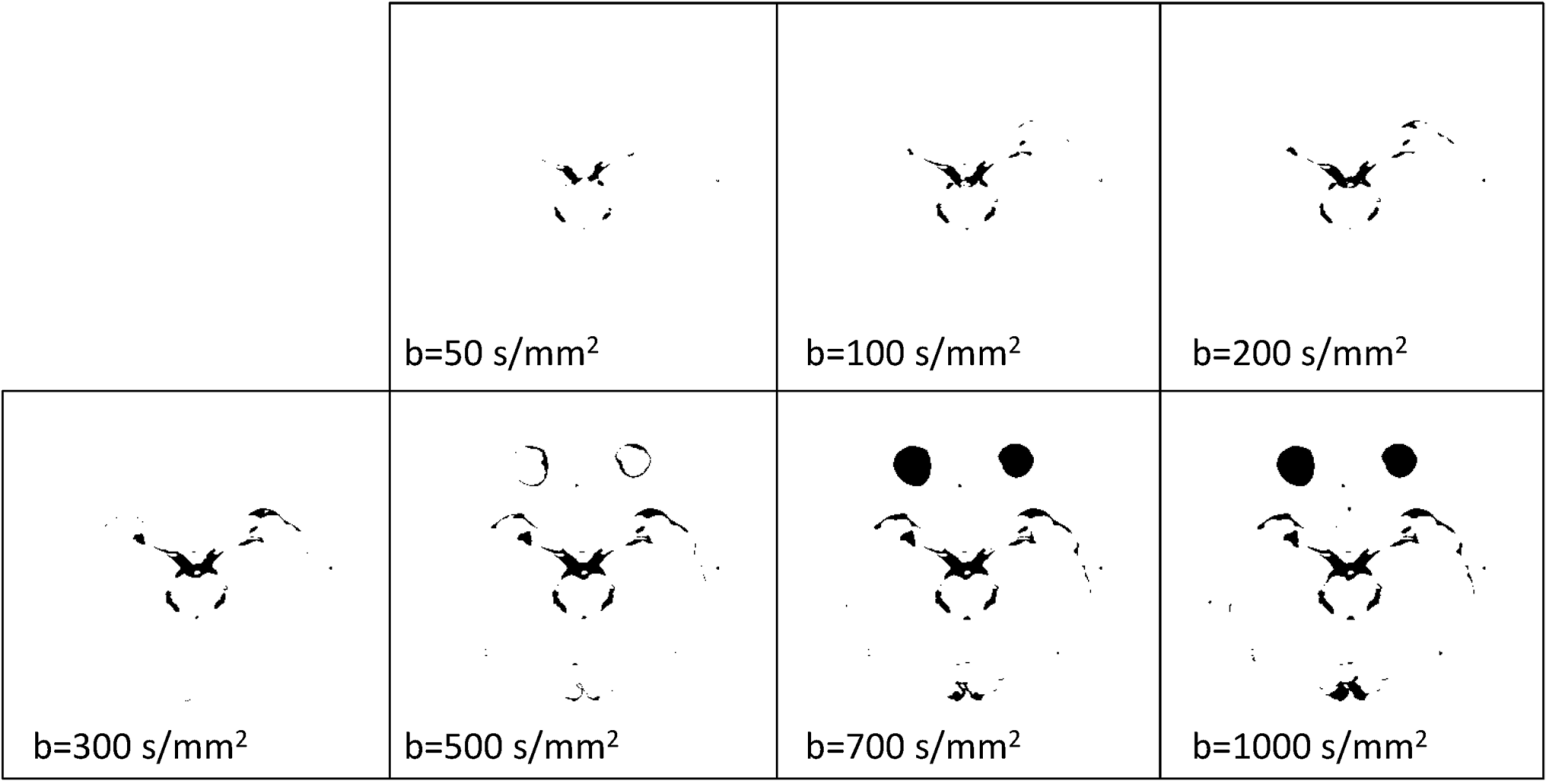

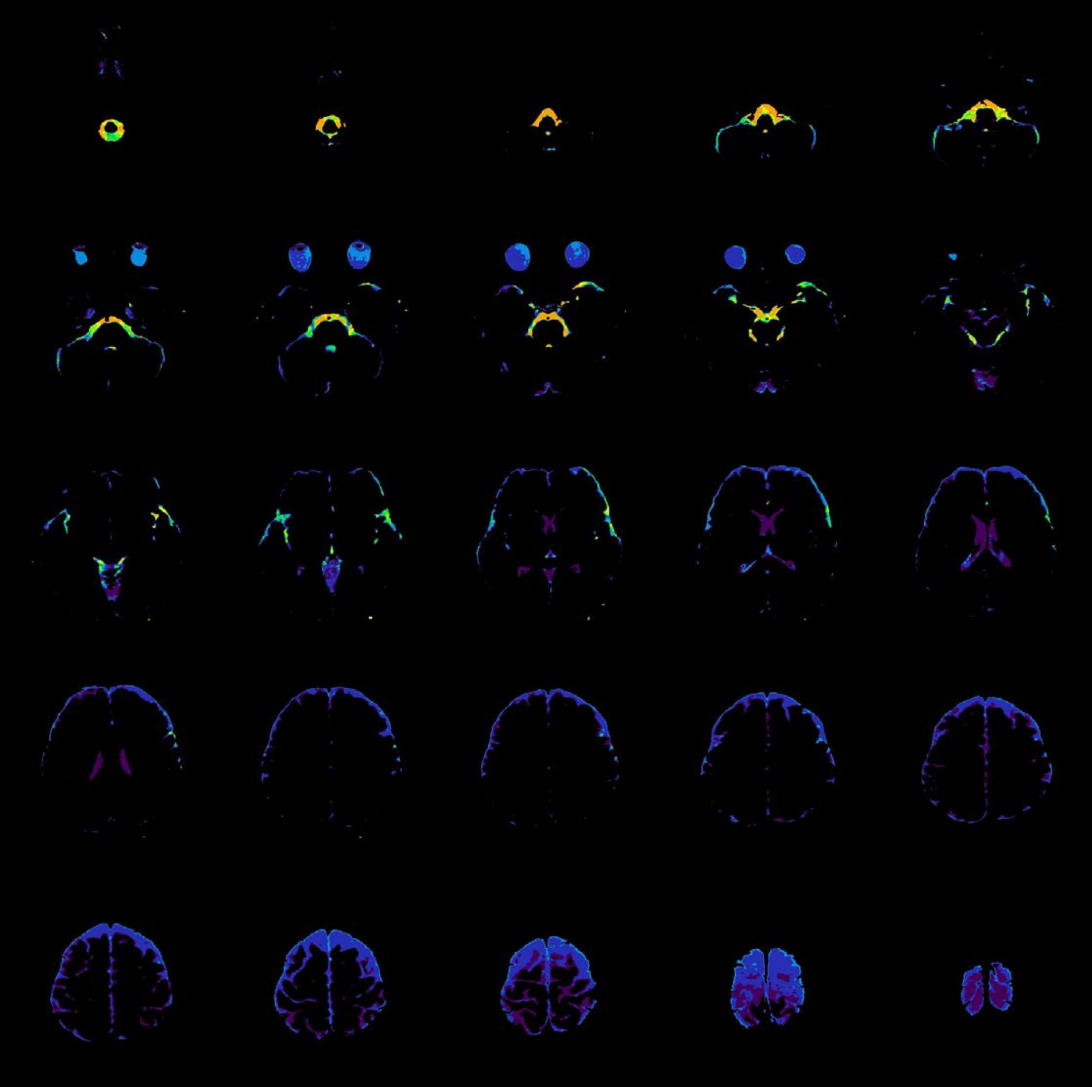
Diffusion analysis of fluid dynamics with incremental strength of motion proving gradient (DANDYISM): **a** binary map of the areas with motion-related signal dephasing with various *b* values; **b** whole-brain color-coded DANDYISM images. Binary map of the areas with motion-related signal dephasing with various b values, which are segmented by the auto-thresholding function of ImageJ software [7]. Images of various *b* values (*b* = 50, 100, 200, 300, 500, 700, 1000 s/mm^2^) at the level of the suprasellar cistern and Sylvian vallecula are shown (**a**). Color composite images in which areas with signal drop due to motion-related signal dephasing on various *b* value images are color-coded with colors as follows: orange: *b* = 50 s/mm^2^, yellow: *b* = 100 s/mm^2^, light green: *b* = 200 s/mm^2^, green: *b* = 300 s/mm^2^, blue: *b* = 500 s/mm^2^, indigo: *b* = 700 s/mm^2^, purple: *b* = 1000 s/mm^2^. Whole-brain images are shown (**b**). Note that signal drop occurs with lower b values in the area including the ventral portion of the posterior fossa, suprasellar cistern, and Sylvian fissure, indicating greater CSF motions. In contrast, signal drop in the lateral ventricle or subarachnoid space of the parietal region occurs only with higher b values, indicating smaller CSF motions

Post-processing, in which DWIs with various b values are composited into a single image to obtain the DANDYISM images, was performed using ImageJ software (U.S. National Institutes of Health, Bethesda, Maryland, USA) [6]. The areas with motion-related signal dephasing were segmented by the auto-thresholding function of ImageJ, which is a variation of the IsoData algorithm (Fig. 2a) [7]. We thus produced color composite images, in which the areas with motion-related signal dephasing on various b value images after the thresholding process, were color-coded as follows: orange: *b* = 50 s/mm^2^, yellow: *b* = 100 s/mm^2^, light green: *b* = 200 s/mm^2^, green: *b* = 300 s/mm^2^, blue: *b* = 500 s/mm^2^, indigo: *b* = 700 s/mm^2^, purple: *b* = 1000 s/mm^2^ (Fig. 2b).

We evaluated the distribution of the CSF area that shows signal drop due to motion-related dephasing using a scoring system (7: more than half the area shows signal drop on a *b* = 50 s/mm^2^ image, 6: signal drop on a *b* = 50 s/mm^2^ image exists in the area, 5: signal drop on a *b* = 100 s/mm^2^ image exists in the area, 4: as previously but for *b* = 200 s/mm^2^, 3: for *b* = 300 s/mm^2^, 2: for *b* = 500 s/mm^2^, 1: for *b* = 700 s/mm^2^, 0: for *b* = 1000 s/mm^2^). A higher score indicates higher CSF motion, and vice versa. Images were evaluated by two neuroradiologists with more than 20 years experience. Any discrepancies between the observers were resolved through discussion, until consensus was reached. Various areas in the cranium were evaluated: the foramen magnum (anterior, posterior), foramen Luschka, foramen Magendie, fourth ventricle (lower, middle, upper), aqueduct, cerebellopontine angle, prepontine cistern (lower, upper), suprasellar cistern, ambient cistern, quadrigeminal cistern, Sylvian vallecula, Sylvian fissure (lower, middle, upper), third ventricle (anterior, posterior), foramen of Monroe, lateral ventricle (anterior horn, body, trigon, inferior horn), interhemispheric fissure (lower, upper), subarachnoid space of the frontal lobe, and subarachnoid space of the parietal lobe. We performed a Tukey test to compare the scores of representative areas (anterior part of foramen magnum, lower part of prepontine cistern, suprasellar cistern, ambient cistern, Sylvian vallecula, middle part of Sylvian fissure, middle part of fourth ventricle, anterior part of third ventricle, foramen Monroe, body of lateral ventricle, parietal subarachnoid space). We also conducted a rank Spearman’s test of the scores and ages in the areas indicated above. The threshold for significant difference was set at *P* < 0.05.

## Results

**Fig. 3:**
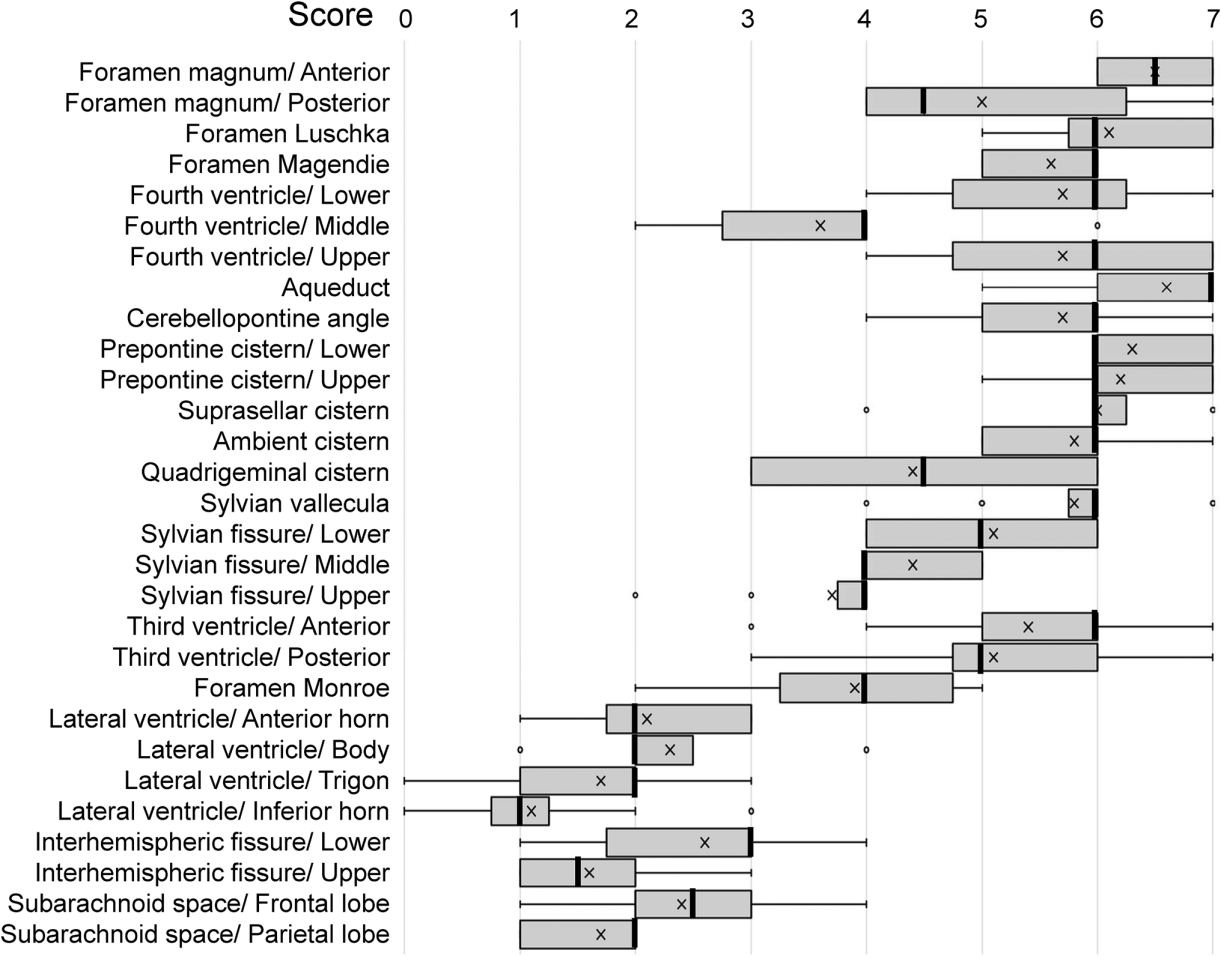
Distribution of scores on the DANDYISM images. Box-and-whisker plots of the CSF scores are shown. Boxes indicate lower and upper quartiles. Thick lines indicate medians. Thin lines indicate minimum and maximum. “*x*”s indicate mean. “*o*”s indicate outliers. A higher score indicates that the signal drop of the CSF takes place with lower b value, indicating greater CSF motions. CSF motions were prominent in the area including the ventral portion of the posterior fossa, suprasellar cistern, and Sylvian fissure, whereas they were smaller in the lateral ventricles and parietal subarachnoid space

**Fig. 4:**
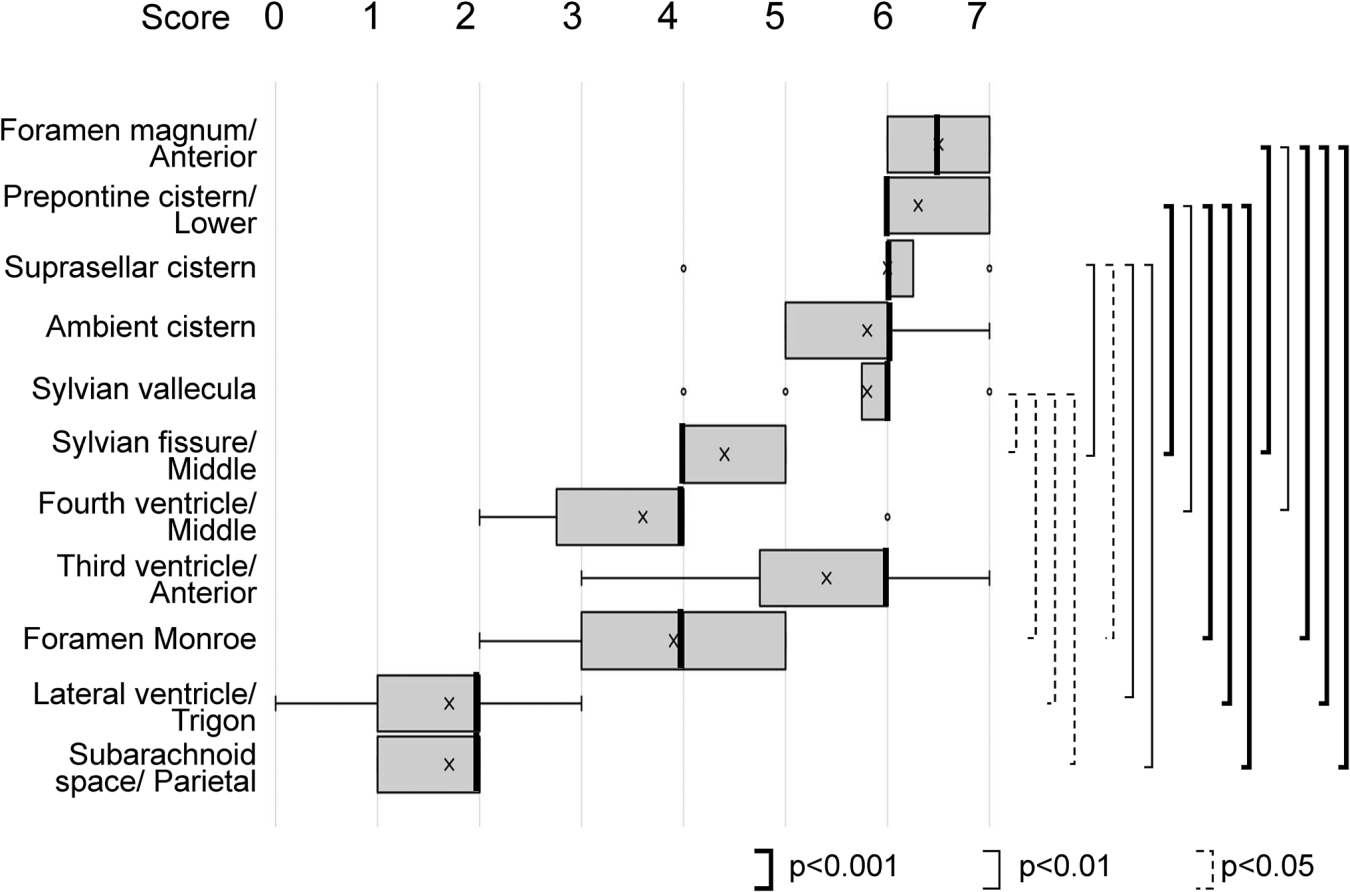
Results of statistical analysis for representative areas. Statistical analysis (Tukey test) was conducted for representative areas (*q*-values for Tukey test are shown in “Appendix”). The area of the ventral portion of the posterior fossa exhibits a statistically significant higher score than that of the ventricles or parietal subarachnoid space

Figure 3 shows the distribution of the scores of all areas evaluated on the DANDYISM images, and Fig. 4 shows the results of the statistical analysis (Tukey test) for the representative areas (*q* values for Tukey test are shown in “Appendix”).

These results indicate that the CSF area in the ventral posterior fossa shows greater movement of CSF (higher scores) compared to other areas. The suprasellar cistern, ambient cistern, and Sylvian vallecula also show greater CSF movement, whereas the CSF within the lateral ventricle shows less motion (lower scores). Moreover, the subarachnoid space of the parietal region shows less motion.

**Fig. 5:**
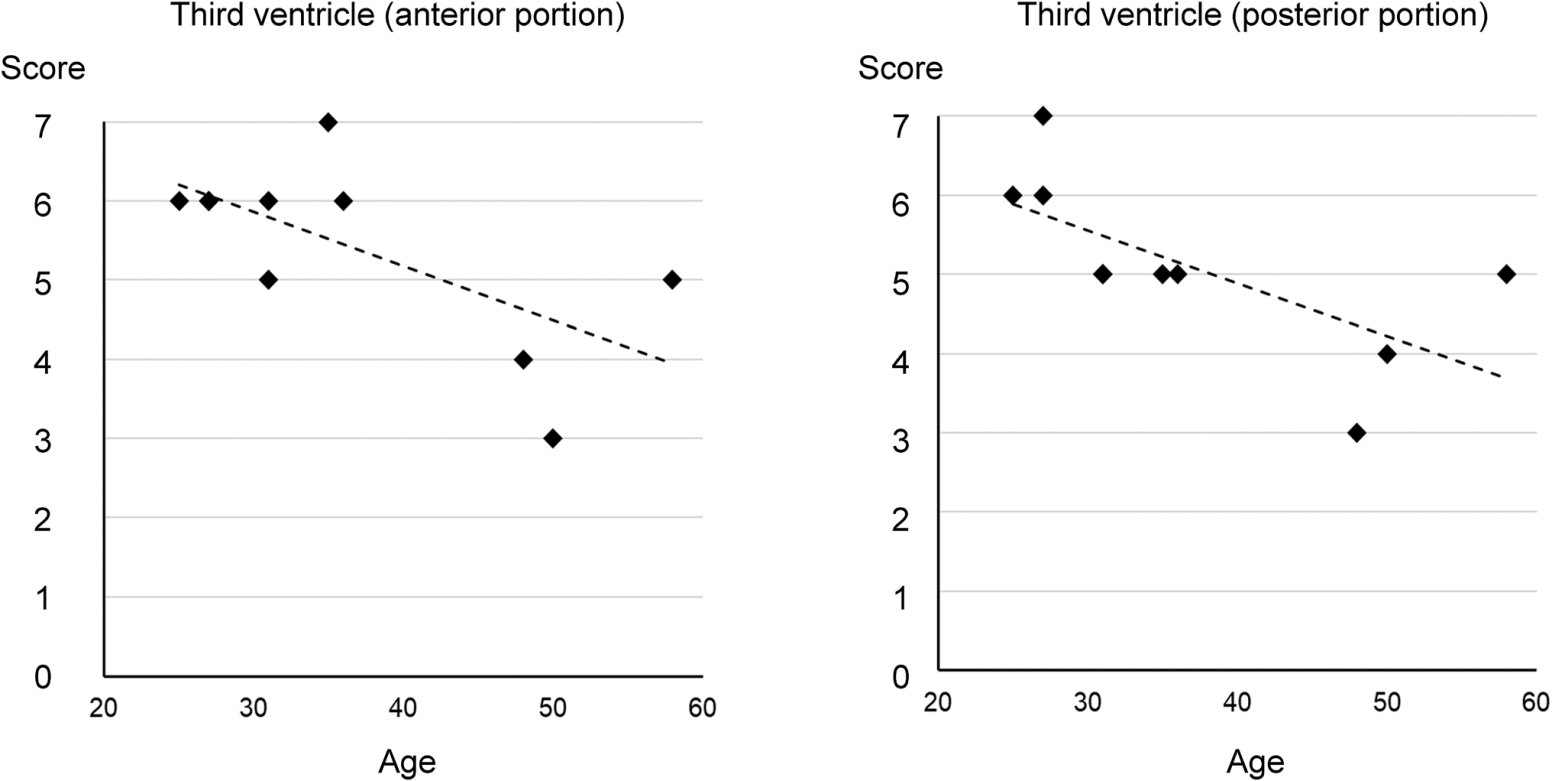
Rank Spearman’s test for scores and ages. The Spearman’s rank correlation coefficients (*r*_s_) between score and age were 0.61 in the anterior portion of the third ventricle (**a**) and 0.81 in the posterior portion of the third ventricle (**b**), showing statistically significant correlation that indicates greater CSF motion in the younger subjects

There was statistically significant correlation between score and age in the anterior (*r*_s_ = 0.61) and posterior portions (*r*_s_ = 0.81) of the third ventricle, indicating greater CSF motion in younger subjects (Fig. 5). However, there was no statistically significant correlation between score and age in any other areas within the cranium.

## Discussion

In a DWI, a moving water molecule exhibits phase shift according to the velocity of the water molecule and length of motion-proving gradient, which can be expressed as *φ* = − *γGvδ*^2^, where φ denotes phase shift; *γ* is gyromagnetic ratio; *G* is gradient strength; *v* is velocity of spin, and *δ* is gradient length. However, this relationship does not hold when the phase shift becomes larger than ± π. In addition, when multiple velocities exist in a single voxel signal, the decrease becomes larger (non-uniform flow effect) [3, 4]. For the purpose of mapping CSF dynamics using DWIs of various b values, we did not attempt to calculate the velocity of the CSF from the datasets of signal with different b values, because the relationship between the velocity and the signal is not uniform. Instead, we mapped the area showing significant signal decrease due to motion-related signal dephasing for each b value, as shown in the DANDYISM images.

The dynamics of CSF have been discussed since a long time [1, 2, 8–12]. In classical theory, CSF is produced actively in the choroid plexus within the ventricles and flows out from the ventricles to be absorbed via arachnoid villi on the surface of the cranium. The choroid plexus is considered to act as a pump in the CSF system. In clinical practice, cases with abnormal ventricular dilatation are not rare, and one of the causes of ventricular dilatation is consider to be an imbalance between CSF production and absorption, for which several mechanisms are responsible. However, the mechanism for hydrocephalus is not fully understood. For example, itiopathic normal pressure hydrocephalus (iNPH) is a type of communicating hydrocephalus for which the pathophysiology is not fully understood; thus, the diagnosis is mainly based on a combination of clinical and imaging findings [13–16]. Several imaging findings have been reported for iNPH in addition to ventricular dilatation, including narrow high-convexity sulci, dilation of the Sylvian fissures, focally enlarged sulci, callosal angle, or periventricular white matter hyperintensities [17, 18]. However, no distinct finding of those mentioned is sufficient for complete diagnosis; thus there have been several attempts to integrate these findings using a scoring method [19, 20].

Although the information for CSF dynamics provided by DANDYISM is mainly on the fast and pulsatile motion of the CSF, the results of the current study provide evidence that casts some doubt on the classical model of CSF dynamics. The CSF motion in the trigon of the lateral ventricle, which is considered to be a site of CSF production in the classical model, exhibited less CSF motion. Regarding absorption, less CSF motion was observed in the subarachnoid spaces in the parietal or frontal area, which are close to the arachnoid granules. In contrast, greater movement of the CSF (higher scores) was observed in the ventral posterior fossa as compared to that in other areas. The suprasellar cistern, ambient cistern, and Sylvian vallecula also exhibited greater CSF movement. These areas contain large arteries including the vertebral arteries, basilar artery, and middle cerebral arteries. The arterial pulsation of these areas may influence the corresponding CSF movement. The findings of this study may, therefore, cast further doubt on the classical model of river-like CSF, which assumes CSF is produced by the choroid plexus in the lateral ventricle and absorbed in the arachnoid granules distributed in the parasagittal area, because the motion of the water molecules is slow in both the ventricle and the subarachnoid space in the frontal and parietal regions.

There are several advantages of DANDYISM method compared to specially designed methods such as the time-spatial labeling inversion pulse (Time-SLIP) or 4D-flow methods. The short acquisition time of DANDYISM method is potentially a great advantage. The acquisition time of the sequence employed in this study was 4 min. As the 4D-flow method uses cardiac gating, the acquisition time tends to be long if attempting to cover the whole brain. Coverage of the whole brain is an additional advantage of this method. The Time-SLIP method can sample only one place within the brain for single evaluation. Therefore, the DANDYISM method is a potentially useful and convenient technique for evaluation of CSF dynamics. As a study for iNPH cases using 4D flow method applied in lower part of the cranium showed significant increase in the CSF stroke volumes at the foramen of Magendie and cerebral aqueduct [21], this DANDYISM method is expected to provide equivalent information for the whole cranium in shorter acquisition time.

The major disadvantage of this DANDYISM method is lack of quantitativeness. It might be possible to calculate the perfusion-related diffusion coefficient (D*) from the signal of *b* = 0 and low b-value diffusion images [22]. However, the signal value varies with the design of the motion-probing gradient or other factors in imaging sequences. It has been reported that CSF pulsation artifacts on apparent diffusion coefficient maps are affected by the number of readout segments [23]. We used a scoring method to evaluate the signal in this retrospective study to avoid influence from the above-mentioned factors. Another disadvantage is that the information of the CSF dynamics provided by DANDYISM is mainly on the fast and pulsatile motion, and the information of the slow and bulk motion of the CSF may not be correctly included.

There are several limitations to the current study. First, as mentioned previously, the DANDYISM is not a quantitative method and, in addition, the chronological aspect cannot be evaluated. Considering that point, this method can be used as a screening method to evaluate the CSF dynamics. The current study did not include subjects with altered CSF dynamics, such as hydrocephalus cases. We will perform further study to apply the DANDYISM method in such cases, in which the results of the current study can be used as control data.

In conclusion, distribution of motion-related signal dephasing by CSF was evaluated on DANDYISM method. The DANDYISM images indicated that CSF motions were prominent in the area including the ventral portion of the posterior fossa, suprasellar cistern, and Sylvian fissure, whereas they were small in the lateral ventricles and parietal subarachnoid space. It was also indicated that CSF motion is correlated with age in the third ventricle. The DANDYISM method may provide information of whole cranial CSF dynamics in the clinical settings within short imaging time.

## Appendix

The result of Tukey test for representative areas.

Tukey test’s *q* values and statistical significances are shown.

**Table:**

	Foramen magnum/anterior	Prepontine cistern/lower	Suprasellar cistern	Ambient cistern	Sylvian vallecula	Sylvian fissure/middle	Fourth ventricle/middle	Third ventricle/anterior	Foramen Monroe	Lateral ventricle/trigon	Subarachnoid space/parietal
Foramen magnum/anterior	–	NS	NS	NS	NS	*P* < 0.01	*P* < 0.01	NS	*P* < 0.01	*P* < 0.001	*P* < 0.001
Prepontine cistern/lower	0.51	–	NS	NS	NS	*P* < 0.01	*P* < 0.01	NS	*P* < 0.01	*P* < 0.001	*P* < 0.001
Suprasellar cistern	1.18	0.67	–	NS	NS	NS	*P* < 0.01	NS	*P* < 0.01	*P* < 0.001	*P* < 0.001
Ambient cistern	1.78	1.26	0.60	–	NS	NS	NS	NS	NS	*P* < 0.01	*P* < 0.001
Sylvian vallecula	1.68	1.18	0.51	0.09	–	NS	NS	NS	NS	*P* < 0.01	*P* < 0.001
Sylvian fissure/middle	4.77	4.27	3.60	3.00	3.09	–	NS	NS	NS	NS	NS
Fourth ventricle/middle	5.73	5.22	4.55	3.96	4.05	0.95	–	NS	NS	NS	NS
Third ventricle/anterior	2.47	1.97	1.30	0.70	0.79	2.30	3.25	–	NS	*P* < 0.01	*P* < 0.01
Foramen Monroe	5.42	4.91	4.24	3.65	3.74	0.64	0.31	2.94	–	NS	NS
Lateral ventricle/trigon	7.40	6.89	6.22	5.63	5.72	2.62	1.67	4.92	1.98	–	NS
Subarachnoid space/parietal	8.07	7.56	6.90	6.30	6.39	3.30	2.35	5.60	2.65	0.67	–

## References

[1] Cushing H (1925). Cameron lectures—delivered at the University of Edinburgh on Oct. 19th, Oct 20th, and Oct 22nd, 1925 LECTURE I.-THE THIRD CIRCULATION AND ITS CHANNELS. Lancet.

[2] Dandy WE (1929). Where is cerebrospinal fluid absorbed?. J Amer Med Assoc.

[3] Le Bihan D, Breton E, Lallemand D, Aubin ML, Vignaud J, Laval-Jeantet M (1988). Separation of diffusion and perfusion in intravoxel incoherent motion MR imaging. Radiology.

[4] Le Bihan D, Breton E, Lallemand D, Grenier P, Cabanis E, Laval-Jeantet M (1986). MR imaging of intravoxel incoherent motions: application to diffusion and perfusion in neurologic disorders. Radiology.

[5] Taoka T, Naganawa S, Kawai H, Nakane T, Murata K (2019). Can low b value diffusion weighted imaging evaluate the character of cerebrospinal fluid dynamics?. Jpn J Radiol.

[6] Schneider CA, Rasband WS, Eliceiri KW (2012). NIH Image to ImageJ: 25 years of image analysis. Nat Methods.

[7] Ridler TW, Calvard S (1978). Picture thresholding using an iterative selection method. IEEETransSyst.

[8] Jost G, Frenzel T, Lohrke J, Lenhard DC, Naganawa S, Pietsch H (2017). Penetration and distribution of gadolinium-based contrast agents into the cerebrospinal fluid in healthy rats: a potential pathway of entry into the brain tissue. Eur Radiol.

[9] Taoka T, Masutani Y, Kawai H, Nakane T, Matsuoka K, Yasuno F (2017). Evaluation of glymphatic system activity with the diffusion MR technique: diffusion tensor image analysis along the perivascular space (DTI-ALPS) in Alzheimer's disease cases. Jpn J Radiol.

[10] Naganawa S, Nakane T, Kawai H, Taoka T (2019). Age dependence of gadolinium leakage from the cortical veins into the cerebrospinal fluid assessed with whole Brain 3D-real inversion recovery MR imaging. Magn Reson Med Sci: MRMS: Off J Jpn Soc Magn Reson Med.

[11] Taoka T, Naganawa S (2018). Gadolinium-based contrast media, cerebrospinal fluid and the glymphatic system: possible mechanisms for the deposition of gadolinium in the brain. Magn Reson Med Sci: MRMS: Off J Jpn Soc Magn Reson Med.

[12] Taoka T, Naganawa S (2020). Glymphatic imaging using MRI. J Magn Reson Imaging.

[13] Irie R, Tsuruta K, Hori M, Suzuki M, Kamagata K, Nakanishi A (2017). Neurite orientation dispersion and density imaging for evaluation of corticospinal tract in idiopathic normal pressure hydrocephalus. Jpn J Radiol.

[14] Yokota H, Vijayasarathi A, Cekic M, Hirata Y, Linetsky M, Ho M (2019). Diagnostic performance of glymphatic system evaluation using diffusion tensor imaging in idiopathic normal pressure hydrocephalus and mimickers. Curr Gerontol Geriatr Res.

[15] Ishii K (2020). Diagnostic imaging of dementia with Lewy bodies, frontotemporal lobar degeneration, and normal pressure hydrocephalus. Jpn J Radiol.

[16] Taoka T, Naganawa S. Imaging for central nervous system (CNS) interstitial fluidopathy: disorders with impaired interstitial fluid dynamics. Jpn J Radiol. 2020. 10.1007/s11604-020-01017-0 PMC7813706 32653987

[17] Kitagaki H, Mori E, Ishii K, Yamaji S, Hirono N, Imamura T (1998). CSF spaces in idiopathic normal pressure hydrocephalus: morphology and volumetry. AJNR Am J Neuroradiol.

[18] Mori E, Kitagaki H (1999). Clinical perspective in normal pressure hydrocephalus. AJNR Am J Neuroradiol.

[19] Elobeid A, Laurell K, Cesarini KG, Alafuzoff I (2015). Correlations between mini-mental state examination score, cerebrospinal fluid biomarkers, and pathology observed in brain biopsies of patients with normal-pressure hydrocephalus. J Neuropathol Exp Neurol.

[20] Kockum K, Lilja-Lund O, Larsson EM, Rosell M, Soderstrom L, Virhammar J, et al. The iNPH Radscale; a radiological scale for structured evaluation of idiopathic normal pressure hydrocephalus. European journal of neurology. 2017. 10.1111/ene.13555 29281156

[21] Yamada S, Ishikawa M, Ito H, Yamamoto K, Yamaguchi M, Oshima M (2020). Cerebrospinal fluid dynamics in idiopathic normal pressure hydrocephalus on four-dimensional flow imaging. Eur Radiol.

[22] Kang KM, Choi SH, Kim DE, Yun TJ, Kim JH, Sohn CH (2017). Application of cardiac gating to improve the reproducibility of intravoxel incoherent motion measurements in the head and neck. Magn Reson Med Sci: MRMS: Off J Jpn Soc Magn Reson Med.

[23] Ohashi T, Naganawa S, Kanou M, Ikeda M (2017). CSF pulsation artifacts on ADC maps obtained with readout-segmented EPI. Magn Reson Med Sci: MRMS: Off J Jpn Soc Magn Reson Med.

